# Facilitating Participation of People with Dementia and their Caregivers in Emergency Care Research

**DOI:** 10.1002/alz.093130

**Published:** 2025-01-09

**Authors:** Clark Benson, Laura M Block, Andrea L Gilmore‐Bykovskyi

**Affiliations:** ^1^ University of Wisconsin Madison School of Medicine & Public Health, Madison, WI USA

## Abstract

**Background:**

People living with dementia (PLWD) are high utilizers of acute illness and emergency care, with over 50% of the more than 6 million people with Alzheimer’s disease and Alzheimer’s disease related dementia (ADRD) visiting an emergency department (ED) annually. While the ED plays an important role meeting the urgent and acute needs of PLWD and their caregivers, presence of ADRD is often not well recognized and ED visits are associated with significant adverse outcomes for PLWD. Despite these factors, research on the emergency care needs of PLWD is extremely limited. Challenges navigating informed consent and assent in the context of a dynamic environment that often necessitates rapid enrollment is a major barrier to conducting ADRD‐focused emergency care research. This presentation reports on a structured framework developed to facilitate participation of PLWD and/or their caregivers in research during ED visits, with attention to maximizing flexible participation and supported decision‐making.

**Method:**

We developed a scenario guided decision‐tree framework for facilitating rapid, flexible enrollment of PLWD and their caregivers in emergency care research. This framework was trialed and iteratively revised through the conduct of a prospective qualitative study (N = 31 participants; 10 PLWD, 15 caregivers, and 3 dyads). This presentation will summarize challenges in facilitating participation, the refined decision‐tree framework, and highlight strategies for successful implementation of the framework.

**Result:**

Major barriers to facilitating participation included navigating supported participation and enrollment in the context of stringent legal and compliance procedures, supporting participation across disease stages, and monitoring for and responding to fluctuations in attention and capacity. The resultant framework guides early assessments to determine tailored procedures for consent, assent, and flexible participation approaches including supported participation.

**Conclusion:**

A major challenge to engaging PLWD in research about their own care experiences is ensuring that research enrollment procedures are responsive to the broad variation in abilities across the continuum of cognitive challenges, decision‐making needs, and participation preferences. The developed framework supports study team members in navigating this challenge in a manner that is inclusive of flexible participation needs and supported as well as proxy decision‐making.

